# *In vivo *imaging of cell behaviors and F-actin reveals LIM-HD transcription factor regulation of peripheral versus central sensory axon development

**DOI:** 10.1186/1749-8104-6-27

**Published:** 2011-05-27

**Authors:** Erica F Andersen, Namrata S Asuri, Mary C Halloran

**Affiliations:** 1 Genetics Training Program, University of Wisconsin, 1117 W. Johnson Street, Madison, WI 53706, USA; 2 Department of Zoology, University of Wisconsin, 1117 W. Johnson Street, Madison, WI 53706, USA; 3 Department of Neuroscience, University of Wisconsin, 1117 W. Johnson Street, Madison, WI 53706, USA

## Abstract

**Background:**

Development of specific neuronal morphology requires precise control over cell motility processes, including axon formation, outgrowth and branching. Dynamic remodeling of the filamentous actin (F-actin) cytoskeleton is critical for these processes; however, little is known about the mechanisms controlling motile axon behaviors and F-actin dynamics *in vivo*. Neuronal structure is specified in part by intrinsic transcription factor activity, yet the molecular and cellular steps between transcription and axon behavior are not well understood. Zebrafish Rohon-Beard (RB) sensory neurons have a unique morphology, with central axons that extend in the spinal cord and a peripheral axon that innervates the skin. LIM homeodomain (LIM-HD) transcription factor activity is required for formation of peripheral RB axons. To understand how neuronal morphogenesis is controlled *in vivo *and how LIM-HD transcription factor activity differentially regulates peripheral versus central axons, we used live imaging of axon behavior and F-actin distribution *in vivo*.

**Results:**

We used an F-actin biosensor containing the actin-binding domain of utrophin to characterize actin rearrangements during specific developmental processes *in vivo*, including axon initiation, consolidation and branching. We found that peripheral axons initiate from a specific cellular compartment and that F-actin accumulation and protrusive activity precede peripheral axon initiation. Moreover, disruption of LIM-HD transcriptional activity has different effects on the motility of peripheral versus central axons; it inhibits peripheral axon initiation, growth and branching, while increasing the growth rate of central axons. Our imaging revealed that LIM-HD transcription factor activity is not required for F-actin based protrusive activity or F-actin accumulation during peripheral axon initiation, but can affect positioning of F-actin accumulation and axon formation.

**Conclusion:**

Our ability to image the dynamics of F-actin distribution during neuronal morphogenesis *in vivo *is unprecedented, and our experiments provide insight into the regulation of cell motility as neurons develop in the intact embryo. We identify specific motile cell behaviors affected by LIM-HD transcription factor activity and reveal how transcription factors differentially control the formation and growth of two axons from the same neuron.

## Background

Neurons must develop characteristic and often complex morphologies to form functional circuits of the nervous system. Development of proper neuronal structure requires precise regulation of multiple cellular processes, including axon formation, outgrowth and branching [1-4]. Axon formation creates a distinct molecular and functional compartment of the neuron and the position of axon initiation is often critical for establishing neuronal polarity [5]. Axons initiate and extend led by highly protrusive growth cones that sense environmental guidance cues. As growth cones advance, the axon shaft undergoes consolidation, a process that suppresses protrusive activity behind the growth cone and maintains axon polarity [1]. Axon branching and/or the extension of multiple axons allow neurons to innervate multiple targets [2,6]. The formation of axon branches in specific locations and prevention of branching at inappropriate sites requires local regulation of signals that promote or suppress protrusions. These processes are critical for neuronal development, yet the mechanisms that regulate axon formation, consolidation and branching in the complex *in vivo *environment remain poorly understood.

Precise regulation of filamentous actin (F-actin) dynamics is essential for the cell motility processes underlying neuronal morphogenesis [1,4,7,8]. A necessary first step in axon formation is accumulation of F-actin at the initiation site, which was first shown *in situ *during axonogenesis in grasshopper sensory neurons [9]. In addition, the organization of F-actin into filopodia is a key prerequisite for neurite formation in cultured mouse cortical neurons [10]. In *Caenorhabditis elegans*, the specific site of F-actin accumulation and axon formation is determined by extracellular UNC-6/netrin, which locally activates a signaling pathway that controls the actin regulatory protein MIG10/lamellipodin [11,12]. In mammalian *in vitro *models of neuronal polarization, actin regulation is also a critical convergence point for polarity signals (reviewed in [13,14]). Nonetheless, our understanding of the mechanisms controlling the process of initial axon emergence from the cell body and the underlying actin rearrangements *in vivo *is far from complete.

During outgrowth, the polarized structure of axons must be established and maintained by consolidation along the axon shaft and restriction of F-actin-based protrusions to the growth cone [1,4]. *In vitro*, consolidation is mediated in part by Rho-kinase inhibition of F-actin at the growth cone neck [15]. Along the axon shaft, calpain proteolytic activity is required to inhibit protrusive activity by reducing levels of cortactin, an actin regulator [16]. Thus, active suppression of F-actin protrusive activity along the axon shaft is required for proper axon extension and to prevent ectopic branching. To date, the process of axon consolidation *in vivo *has not been investigated.

The formation of axon branches also relies on dynamic rearrangements of the actin cytoskeleton [4,6,17-19]. Axon branching can occur by at least two distinct modes: growth cone bifurcation or interstitial branching off the axon shaft [2,6,17]. Although bifurcation is necessary for sensory axon target innervation *in vivo *[20,21], the cytoskeletal rearrangements involved in this process are largely unknown. Interstitial branching appears to be the predominant branching mode in most neuron types studied thus far [2,6,22-24]. During interstitial branching in cultured cortical neurons, F-actin accumulates at branch initiation points and actin polymerization is required for branch formation [25,26]. Branch promoting factors have been shown to act through derepression of calpain-mediated consolidation, thereby releasing the inhibition of F-actin polymerization [16]. Still, little is known about the dynamics of axon branching or the underlying F-actin rearrangements *in vivo*. Studies using live imaging of F-actin *in vitro *have been instrumental to our understanding of axon formation, outgrowth and branching [1,4,15,25,26]. Here, we image F-actin during neuronal morphogenesis in intact embryos, where axons must integrate multiple signals that influence their motility.

Recent studies have brought to light the importance of intrinsic transcription factor regulation in neuronal morphogenesis [27]. Transcription factor expression not only defines neuronal identity, but also can dictate axon trajectory choices downstream of identity decisions. For example, specific combinations of LIM homeodomain (LIM-HD) and homeobox domain (Hox) transcription factors are expressed in subsets of spinal motor neurons, and these determine the motor axon trajectories and innervation of specific muscle targets [28-31]. In retinal ganglion cells, cell-specific expression of Zic2, a zinc-finger transcription factor, and Isl2, a LIM-HD transcription factor, regulate the midline crossing choice of retinal ganglion cell axons at the optic chiasm [32,33]. These transcription factors mediate axon pathway choices at least in part by regulating expression of Eph family axon guidance receptors [30,34,35]. In general, however, the mechanisms by which transcription controls axon motility remain largely unknown.

The zebrafish Rohon-Beard (RB) spinal sensory neurons provide an ideal model to investigate mechanisms controlling neuronal development *in vivo *because of their unique and stereotyped morphology [36,37]. RB neurons extend two types of axons, centrals and peripherals, with distinct trajectories and behaviors [38]. Each neuron extends ascending and descending central axons that grow longitudinally within the spinal cord to form the dorsal longitudinal fasciculus, and a single peripheral axon that exits the spinal cord and branches in the skin. The differential pathways of central and peripheral RB axons are determined in part by differing responses to extracellular signals [38]. Furthermore, LIM-HD transcription factors of the Islet family and their cofactors, CLIMs, are essential intrinsic regulators of RB neuron morphology [39,40]. Ubiquitous expression of a dominant negative CLIM (DN-CLIM) protein disrupts LIM-HD transcriptional activity and results in the reduction or elimination of RB peripheral axons without an apparent effect on the central axons or RB cell fate [39,40]. Thus, these transcription factors are selectively required for peripheral axon formation and/or guidance. In this study, we analyze how LIM-HD transcription factors control cell motility processes and the F-actin cytoskeleton during neuronal morphogenesis *in vivo*.

To investigate the mechanisms controlling RB morphology and to understand how two axons from one cell are guided differently, we used live imaging of axon behaviors and F-actin distribution *in vivo*. We were able to visualize changes in F-actin distribution during dynamic processes such as axon consolidation and branching. We show that extensive F-actin protrusive activity precedes formation of the peripheral axon, and that F-actin concentrates at the site of peripheral axon initiation. In addition, we found that DN-CLIM differentially affects the motility of peripheral and central axons. In most cases, peripheral axons failed to initiate in DN-CLIM-expressing embryos; however, those that formed had significantly reduced growth rates and branching. In contrast, central axon growth rates were faster in DN-CLIM-expressing embryos. Finally, we found that disruption of LIM-HD activity did not affect F-actin-based protrusive activity or the ability of F-actin to accumulate at peripheral axon initiation sites, indicating that F-actin dynamics were unperturbed by DN-CLIM. However, DN-CLIM did influence the location of F-actin accumulation and axon initiation, suggesting LIM-HD activity may function in axon positioning.

## Results

### Live imaging of individual RB neuron development

The RB neurons compose the primary sensory system in the trunk of anamniotic vertebrate embryos [36,41]. RB cell bodies lie in bilateral rows in the dorsal spinal cord and begin extending central axons between approximately 16 and 17 hours post-fertilization (hpf) [37]. Peripheral axons form approximately 2 hours after central axon initiation and continue to extend and branch over a period of several hours. To visualize the axon morphology of individual RB neurons, we used a transient mosaic cell labeling approach [42]. We injected plasmid DNA encoding a membrane-targeted fluorophore (GFP-CAAX or TagRFP-CAAX) driven by a *cis*-regulatory element from the *neurogenin1 *gene (*-3.1 ngn1*) [43] (see Materials and methods). This method allows visualization of the entire neuron structure during all stages of development.

**Figure 1:**
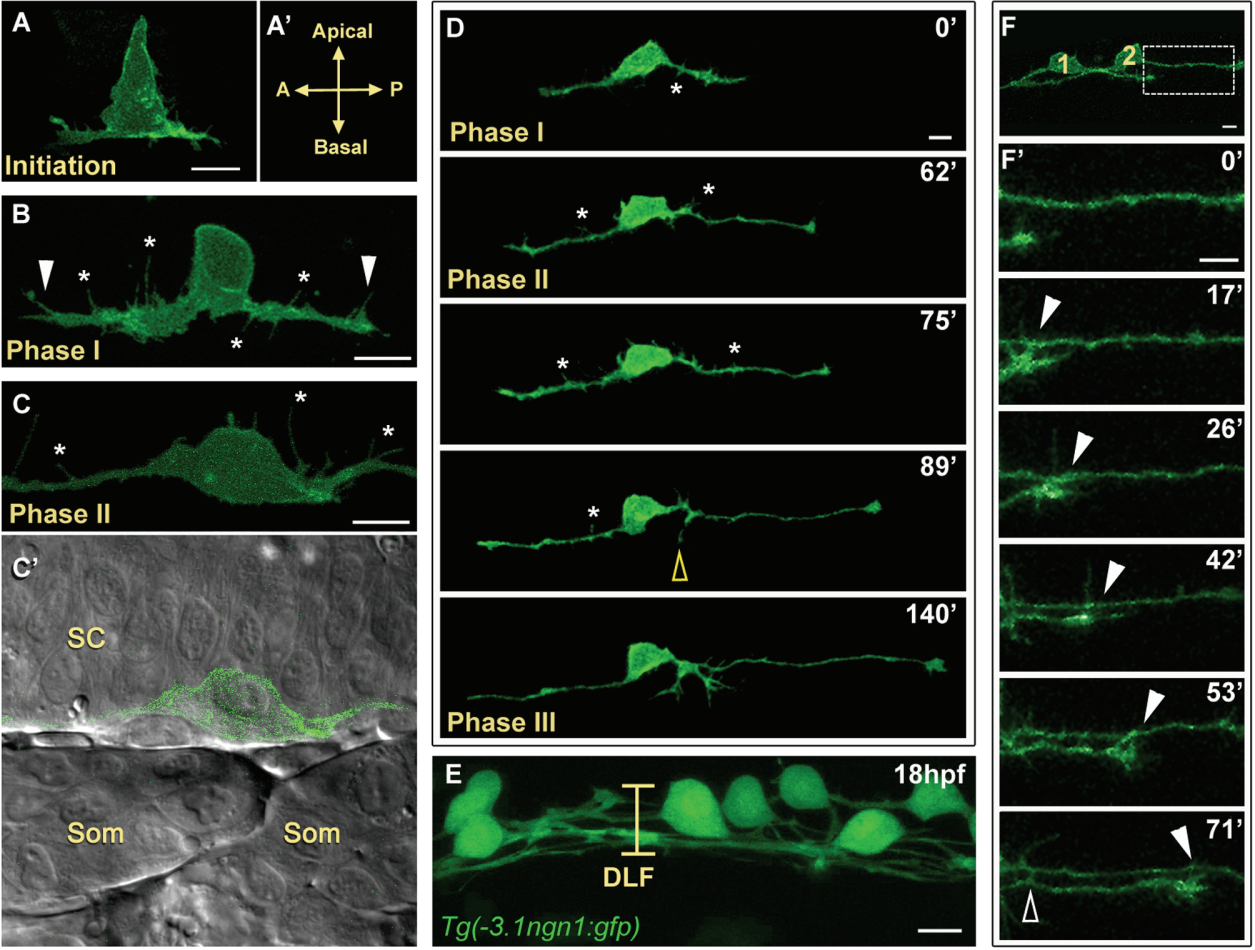
**Central axon development in RB neurons**. All images are dorsal-lateral views with anterior to the left and are confocal projections unless noted. Individual RB neurons labeled by transient mosaic expression of TagRFP-CAAX (pseudocolored green) (A-D) or GFP-CAAX (F) in wild-type embryos. **(A) **Central axon initiation. Central axons form at the basal surface of the cell body at the anterior (A) and posterior (P) sides of the cell. **(A') **Indicates orientation. **(B) **RB neuron in phase I of central axon outgrowth. RB has thick central axons with many filopodial protrusions (asterisks) along the axon shafts and growth cones (arrowheads). **(C,C') **RB neuron in phase II of central axon outgrowth. (C) The central axon shafts display transient filopodial protrusions (asterisks). Growth cones have extended out of the field of view. (C') Single XY plane (green and DIC channel overlay) of RB in (C). The central axons grow along the edge of the spinal cord (SC) neuroepithelium. Som, somites. **(D) **Selected images from an approximately 3.5-hour time-lapse of RB central axon outgrowth, phases I to III. The peripheral axon initiates during phase II (yellow open arrowhead). Asterisks indicate transient filopodial protrusions during phases I and II. See Additional file 1 for a movie. **(E) **Image showing all RB neurons labeled in a transgenic *Tg(-3.1ngn1:gfp) *embryo. At 18 hpf, the dorsal longitudinal fascicle (DLF) is loosely bundled (bracket). (F,F') Central axon fasciculation. **(F) **Two labeled RB neurons (1 and 2) have begun to extend central axons. Box indicates area shown in (F'). **(F') **Time-lapse of RB central axon fasciculation starting at 17 hpf. RB1 growth cone contacts RB2 axon as it extends (arrowheads) and RB1 axon makes transient lateral filopodial contacts (open arrowhead). Time is displayed in minutes. Scale bars = 10 μm.

We first characterized cell behaviors of developing RB neurons in wild-type embryos. During early developmental stages, RB cell bodies have a prominent asymmetric morphology, with tapered apical and broad basal surfaces (Figure 1A). The ascending and descending central axons initiate outgrowth simultaneously from opposite ends at the basal side of the cell body (Figure 1A,B) and extend along the edge of the spinal cord neuroepithelium (Figure 1C,C'), where they pioneer the dorsal longitudinal fasciculus. Central axons grow steadily, without substantial pausing or retracting. Nonetheless, we defined three distinct phases (I, II, and III) of central axon outgrowth based on distinctive morphological features and behaviors (Figure 1).

During their initial outgrowth (phase I), within approximately 25 μm from the cell body, the central axons are thick and display protrusive activity along the entire length of the axon shaft (Figure 1B,D). The central growth cones extend relatively slowly during this phase (mean phase I extension rate = 12.8 ± 2.1 μm/h). In the second phase of central axon outgrowth, when axons are between approximately 25 and 50 μm long, membrane protrusions along the proximal axons are reduced and the axons display a more polarized morphology, with consolidated axon shafts and distinct protrusive growth cones (Figure 1C,D; Additional file 1). This morphological transition is accompanied by an increase in extension rates (mean phase II rate = 23.1 ± 1.1 μm/h). During this phase, central growth cones come into contact with axons from neighboring RB neurons and grow along them to form the dorsal longitudinal fasciculus (Figure 1E,F). In addition, transient filopodial protrusions continue to form along the axon shaft during this phase, and these can make lateral contact with other central axons (Figure 1F), suggesting they could mediate recognition and fasciculation. In the third phase, after central axons have reached a length of 50 μm or greater, their growth cones extend more rapidly (mean phase III rate = 50.3 ± 2.1 μm/h). Although filopodial protrusions continue to form along the central axons in phase III, their frequency in general decreases in proportion to increasing central axon length (Figure 1D).

Formation of the peripheral axon is a particularly important step of RB development because it is the point at which discrete central and peripheral compartments with different axon trajectories and behaviors are established. The peripheral axon forms orthogonally to the central axons, exits the spinal cord, and grows into a very different environment than the central pathway. Peripheral axons initiate during phase II or III of central axon outgrowth (Figure 1D), and become established during phase III. Single cell labeling allowed us to determine the site of peripheral axon formation. Like the central axons, the peripheral axon forms at the basal edge of the RB neuron; however, the precise anterior-posterior site at which it forms is variable from cell to cell. In 40% of cells (n = 47), the peripheral axon emerged directly from the cell body, and in the other 60% it arose as a branch from one of the central axons. The branches did not form preferentially from either the ascending or descending central axon, but initiated from both axons in equal proportions. However, in the majority of neurons (83%), the peripheral axon initiation site was restricted to a region near the cell body. On average, the distance from the cell body center to the peripheral axon initiation site was 10.7 ± 0.67 μm (approximately one cell body diameter). Thus, it appears there is a specific compartment of the RB cell that is capable of generating a peripheral axon.

**Figure 2:**
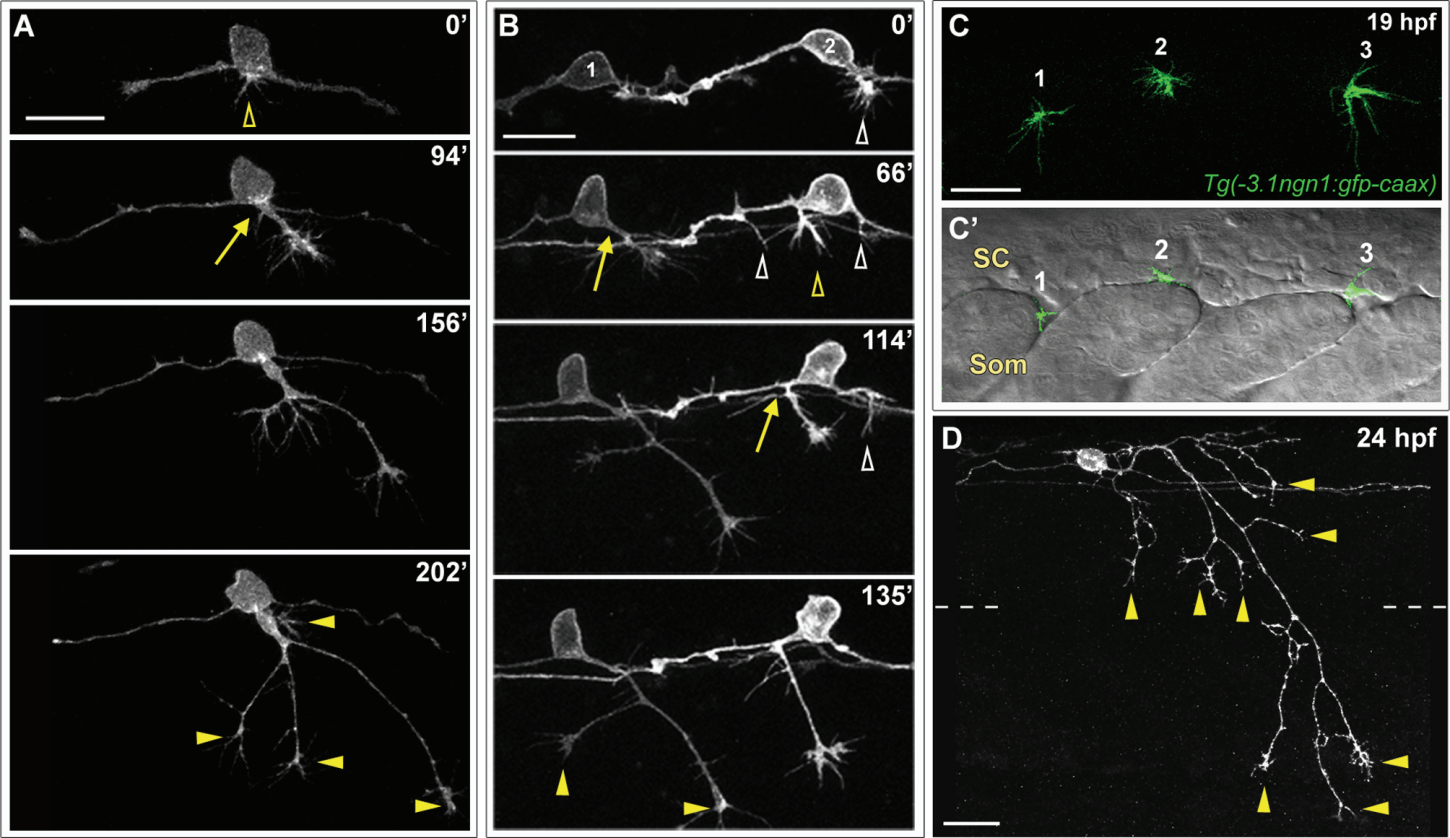
**Peripheral axon development in RB neurons**. **(A,B,D) **Individual RB neurons labeled by transient mosaic expression of TagRFP-CAAX (A,D) or GFP-CAAX (B) in wild-type embryos. Dorsal-lateral views, anterior is left. Images are confocal projections. (A) Time-lapse of RB peripheral axon outgrowth from the cell body. Several filopodial protrusions occur in the cell body region during peripheral initiation (yellow open arrowhead). The peripheral axon forms off the cell body (yellow arrow), exits the spinal cord (SC) and grows in a ventral direction while branching (yellow arrowheads indicate branch tips) in the epidermis. (B) Time-lapse of two RB neurons (1 and 2) showing peripheral axon emergence as central axon branches. RB1: a peripheral axon initiates off the descending central axon (yellow arrow), and grows ventrally while extending branches (yellow arrowheads). RB2: multiple neurites initiate as branches off both ascending and descending central axons (open arrowheads); however, only one becomes an established peripheral axon (yellow open arrowhead and arrow), and the others retract (white open arrowheads). See Additional file 2 for movie. Time displayed in minutes (A,B). **(C,C') **Lateral views of a 19-hpf transgenic *Tg(-3.1ngn1:gfp-caax) *embryo showing the anterior-posterior position where peripheral axons exit the SC. (C) Confocal projection (green channel) of four 1-μm Z-planes showing newly formed peripheral growth cones of three RB neurons (1, 2, and 3). (C') Single XY plane (green and DIC channel overlay) reveals sites of peripheral axon emergence. Growth cones 1 and 3 extend between somites (Som). Growth cone 2 grows over the somite. (D) Example of mature RB neuron morphology in a 24-hpf wild-type embryo. RB has an elaborated peripheral axon with many branches (some indicated with yellow arrowheads). Dashed lines indicate the position of the horizontal myoseptum. Scale bars = 25 μm.

Each RB neuron extends a single peripheral axon to the skin, yet the mechanisms limiting peripheral axon number are not understood. While the majority of our time-lapse experiments showed a peripheral axon initiate and extend from one location (Figure 2A), in some cases (24%, n = 21 neurons) we observed multiple short neurites initiating in different locations of the cell at the same time (Figure 2B; Additional file 2). Eventually one of these exited the spinal cord and became established as the successful peripheral axon while the others retracted. These behaviors suggest that a mechanism exists to suppress supernumerary peripheral axons after one becomes stabilized and successfully exits the spinal cord. Furthermore, we found that peripheral axons frequently exit the spinal cord at somite boundaries (67%, n = 36 neurons; Figure 2C), suggesting the environment between somites is favorable for outgrowth. Together, these data suggest the location of peripheral axon formation may be influenced by a combination of intrinsic bias to a compartment including the basal cell body and proximal central axons, and environmental signals or spatial availability at the somite boundaries.

**Figure 3:**
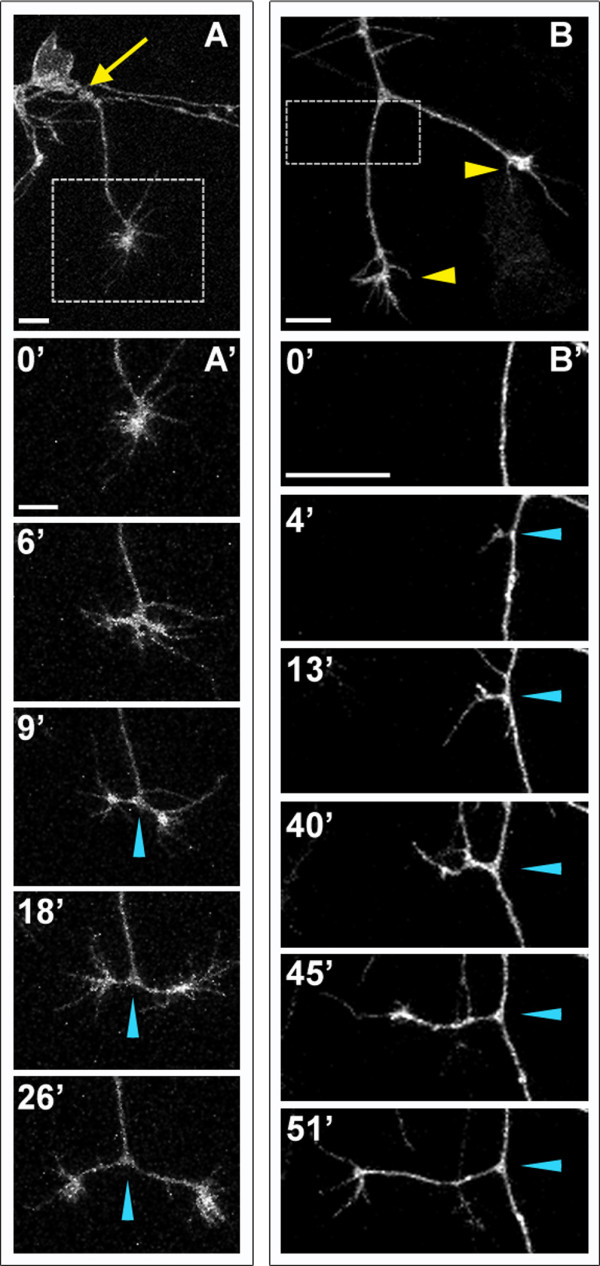
**Peripheral axons branch by bifurcation and interstitial branching**. Individual RB neurons labeled by transient mosaic expression of GFP-CAAX in wild-type embryos. Lateral views, anterior is left. Images are confocal projections. **(A) **An unbranched peripheral axon formed at a site posterior to the RB cell body (yellow arrow). Box shows area imaged in (A'). **(A') **Time-lapse of peripheral axon bifurcation (blue arrowheads indicate branch point). **(B) **A branched peripheral axon with growth cones extending ventrally (yellow arrowheads). Box shows area imaged in (B'). **(B') **Time-lapse of interstitial branch formation (blue arrowheads indicate branch point). Time is displayed in minutes. Scale bars = 10 μm.

Peripheral RB axons undergo extensive secondary branching to form complex arbors in the skin (Figure 2D). Live imaging revealed that branches form continuously during peripheral axon outgrowth, by both bifurcation and interstitial branching mechanisms (Figure 3). As peripheral axons exit the spinal cord, their growth cones become very large and the axons begin branching. The first branching event generally occurs by growth cone bifurcation (Figure 3A). Subsequent branches form by a combination of both branching modes occurring simultaneously. We also imaged the spatio-temporal dynamics of interstitial branching and found that formation of an interstitial branch begins with extension of a filopodium, which becomes a growth cone-tipped neurite (Figure 3B), similar to branching seen in culture [25,26] and in other neuron types *in vivo *[19,23,44-47].

### Imaging F-actin distribution during RB development

Precise regulation of the actin cytoskeleton is essential for axon formation, guidance and branching, yet little is known about the dynamics of actin rearrangements during these events *in vivo*. To image F-actin distribution during RB development, we used a biosensor containing the calponin homology domain of utrophin fused to mCherry (mCherry-UtrCH), which selectively labels F-actin without affecting its dynamics *in vivo *[48]. Transient mosaic expression of mCherry-UtrCH behind the *-3.1ngn1 *RB driver allowed us to visualize the dynamic remodeling of F-actin in RB neurons *in vivo *(see Materials and methods). Moreover, we found that neurons expressing UtrCH developed normally and their axon extension rates did not differ significantly from neurons expressing fluorophores alone (Table 1), indicating that the reporter does not interfere with actin dynamics.

**Table 1:** Motility comparison between control neurons expressing a fluorophore only versus neurons expressing the UtrCH F-actin reporter

	Average central growth rate (μm/h)	Average peripheral growth rate (μm/h)	Average number of filopodia per hour
Fluorophore only	23.8 ± 1.4 (n = 8)	40.9 ± 2.7 (n = 19)	40.9 ± 2.4 (n = 8)
mCherry-UtrCH	22.4 ± 1.8 (n = 8)	40.6 ± 2.5 (n = 7)	37.1 ± 2.3 (n = 9)
*P*-value	0.55	0.95	0.28

*P*-values are based on two-tailed Student's *t*-test comparing fluorophore controls and UtrCH-expressing cells for each measurement.

**Figure 4:**
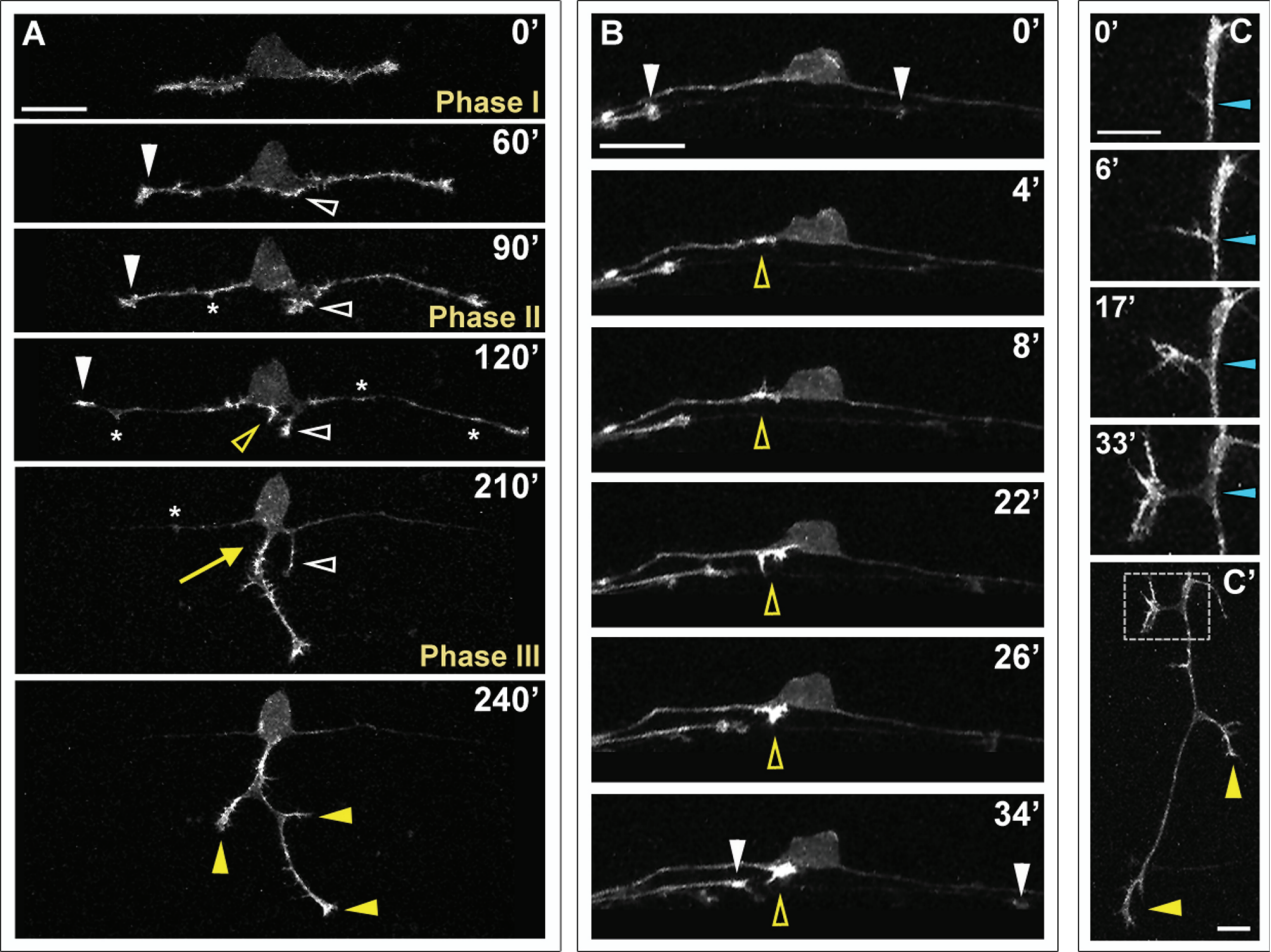
**F-actin distribution in developing RB neurons**. F-actin labeled in individual RB neurons by transient mosaic expression of mCh-UtrCH in wild-type embryos. Dorsal-lateral views, anterior is left. Images are confocal projections. **(A) **Time-lapse showing F-actin distribution during RB neuron development. F-actin is concentrated in the growth cones of central axons (white arrowheads) and peripheral axon branches (yellow arrowheads) as they extend. Accumulation of F-actin occurs at the basal edge of the cell body and becomes concentrated at the distal tip of a neurite that initiates posterior to the cell body and later retracts (white open arrowheads). A second neurite initiates directly off the cell body (yellow open arrowhead) and becomes established as the peripheral axon (yellow arrow). F-actin signal is distributed along the central axon shafts during phase I, but is diminished in the axon shaft during phases II and III. Transient F-actin patches occur along the central axon shafts during phases II and III (some indicated with asterisks) and decrease in frequency over time. See Additional file 3 for movie. **(B) **Time-lapse showing F-actin accumulation during peripheral axon initiation (open yellow arrowheads). White arrowheads indicate central axon growth cones of neighboring labeled RB neurons extending through the field of view. **(C) **Time-lapse showing F-actin dynamics during interstitial branch formation in a peripheral axon. F-actin accumulates in a filopodium (0', 6') then concentrates in the growth cone of the extending branch (17', 33'). Blue arrowheads indicate branch point. **(C') **Low magnification view of branched peripheral axon at the end of the time-lapse. Box indicates region shown in (C). Yellow arrowheads indicate peripheral growth cones. Time is displayed in minutes. Scale bars in (A,B) = 25 μm, in (C) = 10 μm.

We imaged F-actin during several processes of RB development. During the first phase of central axon outgrowth, F-actin signal is strong in the growth cones and also in growth cone-like filopodial and lamellipodial protrusions along the central axons (Figure 4A; Additional file 3). This result indicates that these early axons are not fully consolidated and might explain their slower growth rates. During phase II, the F-actin signal remains concentrated in the growth cones but diminishes along the axon shaft as it undergoes consolidation. Scattered F-actin patches and F-actin rich filopodia continue to form along the central axons and cell body, but become less frequent as the central axons extend. During peripheral axon formation, F-actin accumulates at the site on the cell body (Figure 4A) or central axon (Figure 4B) where the peripheral axon initiates. Strong F-actin signal remains in the peripheral growth cone as it extends out of the spinal cord, enlarges, and begins to branch (Figure 4A). During interstitial branch formation, F-actin becomes enriched in the initial filopodium and localizes to the periphery of the new growth cone as it enlarges and extends (Figure 4C). This result is consistent with observations of F-actin accumulation at interstitial branch points prior to branch formation in cultured cortical neurons [26].

### Imaging RB development in embryos with disrupted LIM-HD transcription factor activity

**Figure 5:**
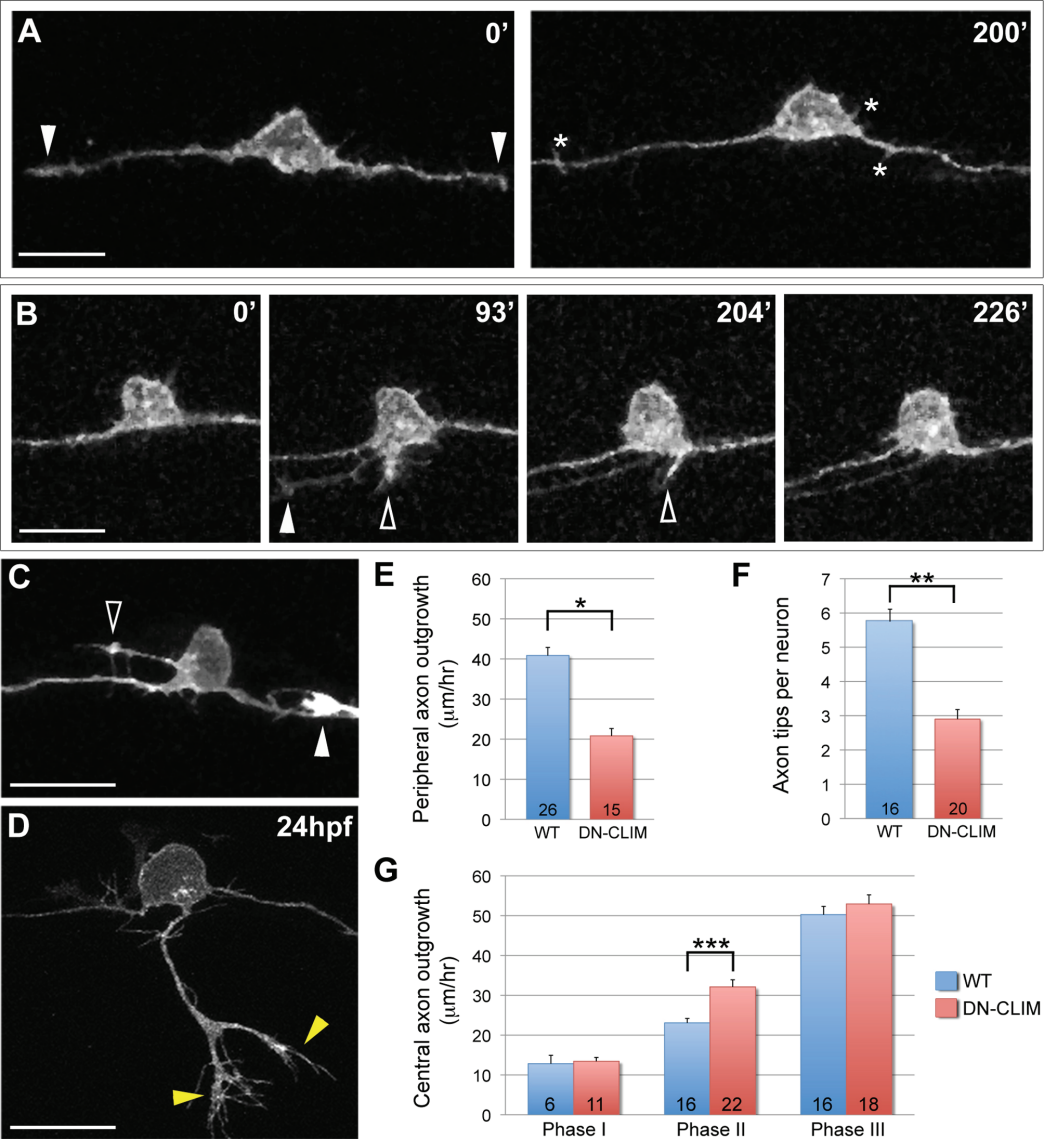
**Effects of LIM-HD disruption on RB axon development**. **(A-D) **Individual neurons labeled by transient mosaic expression of TagRFP-CAAX in DN-CLIM-expressing embryos. Dorsal-lateral views, anterior is left. Images are confocal projections. (A) Two images from a time-lapse in a DN-CLIM embryo. Arrowheads indicate central growth cones at 0'. No peripheral axons initiate. Transient filopodia form off cell body and axon shafts (asterisks). See Additional file 4 for movie. (B) Time-lapse in a DN-CLIM-expressing embryo. A peripheral axon initiates, but fails to extend. Transient neurites form in two locations (open arrowheads) and subsequently retract. Arrowhead at 93' indicates growth cone of neighboring central axon growing through the field of view. (C) Ectopic neurite formation in a DN-CLIM-expressing embryo. A long filopodial protrusion extends from an ectopic apical location on the cell body (open arrowhead). The arrowhead indicates the growth cone of neighboring central axon that enters the field of view. (D) Example of a peripheral axon in a 24-hpf DN-CLIM-expressing embryo. The axon is shorter and less branched than the wild type (compare to Figure 2D; horizontal myoseptum in Figure 5D is out of frame). Yellow arrowheads indicate branches. See Additional file 5 for movie. (A-D) Time displayed in minutes. Scale bars = 25 μm. **(E) **Quantification of average central axon extension rates in wild-type (WT) versus DN-CLIM-expressing embryos. Central axon extension rates are significantly faster in DN-CLIM embryos during phase II. **(F,G) **Quantifications of average peripheral axon extension rates (F) and average number of branches per peripheral axon (G) in wild-type versus DN-CLIM-expressing embryos. *P < 10^-7^, **P < 10^-6^, and ***P = 0.0004 (two-tailed *t*-test). Error bars represent standard error of the mean. Numbers in bars signify number of neurons.

Previous studies revealed that the activity of LIM-HD transcription factors is required for establishment of proper RB morphology [39,40]. Expression of DN-CLIM results in the reduction or elimination of RB peripheral axons without an apparent effect on central axons. However, the mechanisms by which these transcription factors regulate peripheral axon outgrowth and motile axon behaviors are not known. We sought to determine whether the DN-CLIM phenotype results from an inability to form filopodial protrusions associated with peripheral axon initiation, an inability to stabilize these protrusions and convert them to axons, or a failure of the axons to exit the spinal cord. To test these possibilities, we imaged individually labeled RB neurons in DN-CLIM-expressing embryos during the time period when peripheral axons normally would form. In the majority of neurons (68%, n = 25 neurons), the peripheral axon failed to initiate, yet protrusive activity along the central axons appeared normal (Figure 5A; Additional file 4). This result suggests DN-CLIM does not affect the formation of filopodia that precede peripheral axons, but may inhibit their stabilization and transition into axons. Indeed, in some cases (20% of neurons), neurites resembling initiating peripheral axons formed orthogonally to the central pathway but failed to become established and extend out of the spinal cord (Figure 5B). A few of these neurites formed at ectopic locations along the apical-basal axis of the cell body (three of five neurons; Figure 5C), suggesting that LIM-HD transcription factor activity may also regulate axon positioning in RB neurons. A small proportion of RB neurons (12%) in DN-CLIM-expressing embryos extended a peripheral axon that exited the spinal cord and grew along the correct pathway to the skin. However, these axons had aberrant morphology (Figure 5D, compare to Figure 2D; Additional file 5), and displayed significantly slower rates of extension (Figure 5E) and fewer branches (Figure 5F) than wild-type peripheral axons, indicating that the dynamics of peripheral outgrowth and branching are disrupted by DN-CLIM. Together these results suggest that DN-CLIM primarily affects peripheral axon initiation, but also influences other cell motility processes required for peripheral axon extension and the ability to form secondary branches in the periphery.

Previous studies examining LIM-HD regulation of RB development concluded that DN-CLIM did not affect RB central axons [39,40]. However, our live imaging showed that central axon growth rates were in fact affected in DN-CLIM-expressing embryos. We discovered that during phase II of central axon extension, when peripheral axons normally initiate outgrowth, central axon growth rates are significantly faster in DN-CLIM-expressing embryos (Figure 5G). These results indicate that DN-CLIM has specific, opposite effects on the motility of peripheral versus central axons. Moreover, these data argue that the effects of DN-CLIM on peripheral axon outgrowth are not simply due to a developmental delay or to poor health of the neurons.

### Imaging F-actin distribution during RB development in embryos with disrupted LIM-HD transcription factor activity

**Figure 6:**
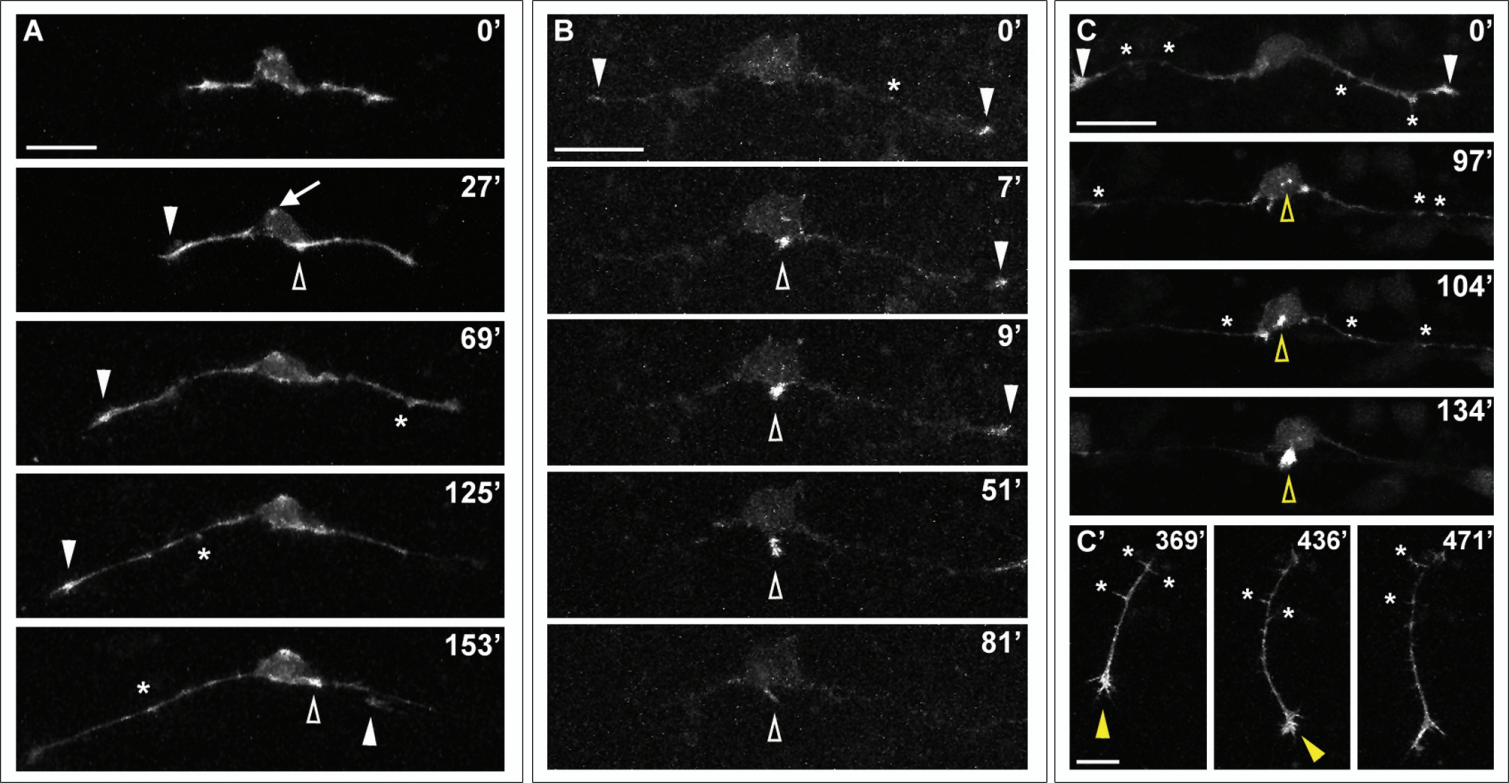
**Effects of LIM-HD disruption on F-actin distribution in developing RB neurons**. Time-lapse sequences of F-actin distribution in DN-CLIM-expressing embryos. F-actin is labeled by transient mosaic expression of mCh-UtrCH. Dorsal-lateral views, anterior is left. Images are confocal projections. **(A) **Neuron in which no peripheral axon initiates. F-actin accumulates transiently near the cell body (open arrowheads) and in patches along central axons (some indicated with asterisks). F-actin also localizes to central growth cones (white arrowheads; including neighboring central growth cone extending through field at 153'). Ectopic F-actin signal occurs at the apical edge of the cell body (example indicated with arrow at 27'). See Additional file 6 for movie. **(B) **Neuron in which peripheral neurite with strong F-actin signal initiates from the cell body (open arrowhead), but retracts. F-actin is also visible in central growth cones (arrowheads) and transiently along the central axon shaft (example indicated with asterisk). See Additional file 7 for movie. **(C,C') **Neuron in which peripheral axon initiates and extends into the periphery. (C) F-actin accumulation is visible in growth cones of central axons (arrowheads), which extend out of the field of view, and transiently along the central axon shaft (some indicated with asterisks). Accumulation of F-actin occurs in an ectopic apical location of the cell body and becomes concentrated at the initiating neurite tip (open yellow arrowheads). (C') Shifted focal plane shows established peripheral axon extending ventrally without branching (yellow arrowhead indicates growth cone). Asterisks indicate some F-actin-rich filopodial protrusions along the peripheral axon shaft. Time is displayed in minutes. Scale bars in (A-C) = 25 μm, in (C') = 10 μm.

We also examined the effects of LIM-HD disruption on actin dynamics, by imaging F-actin in DN-CLIM-expressing embryos. We still detected F-actin accumulations in growth cones and transient filopodial protrusions along the central and peripheral axons despite the fact that peripheral axons either failed to form or had aberrant outgrowth behavior (Figure 6). In neurons that failed to initiate a peripheral axon, we observed transient accumulations of F-actin at the basal cell body or proximal central axon, where peripheral axons would normally initiate (Figure 6A; Additional file 6). Moreover, in neurons that formed either transient (Figure 6B; Additional file 7) or successful (Figure 6C) peripheral axons, strong F-actin signal was detected in the initiating process. We quantified protrusive activity during phase II of central axon growth by measuring the number of F-actin-containing filopodia that form, and found no significant difference between wild-type and DN-CLIM-expressing embryos (average number of filopodia/h = 37.1 ± 2.3 in wild type (n = 9) versus 33.8 ± 1.9 in DN-CLIM (n = 9); *P *= 0.28). We also observed ectopic accumulations of F-actin in more apical regions of the cell body (Figure 6A,C) in some DN-CLIM-expressing neurons (three of ten neurons compared to zero of ten in wild-type), which supports the idea that DN-CLIM may have a more general effect on cell polarity or axon positioning. Together, these results suggest that signals downstream of LIM-HD transcription factors are not required for initial F-actin accumulation, but may instead regulate another aspect of motility that underlies peripheral axon formation.

## Discussion

### Axon behaviors and F-actin dynamics in wild-type RB neurons

In this study, we were able to directly visualize F-actin during axon development *in vivo *using the UtrCH biosensor. This reporter has been shown to bind selectively to F-actin without stabilizing it [48], and has revealed F-actin dynamics during multiple cell motility processes, including wound healing and cytokinesis in *Xenopus *embryos [49,50], neural crest cell or neutrophil migration in zebrafish [51,52], and growth cone turning in cultured neurons [53]. Here we show that neural-specific expression of UtrCH provides excellent spatial-temporal resolution of F-actin during axon development *in vivo*. Moreover, the UtrCH probe did not disrupt actin-based neuronal motility when expressed mosaically under control of the *-3.1ngn1 cis*-regulatory element. Our experiments provide the first characterization of F-actin dynamics during axon consolidation, initiation, and branching *in vivo*.

Consolidation is essential for establishing and maintaining axon shape and polarity, yet how this process occurs *in vivo *is not understood. Live imaging of newly forming RB central axons revealed that these axons undergo a dynamic transition in their consolidation state during the first few hours of outgrowth. Our results suggest that consolidation and acquisition of polar axon morphology are not immediate processes but occur gradually as axons extend. Furthermore, our finding that transient F-actin-based filopodia continue to form along the axon shaft after many hours suggests RB axons do not become completely consolidated in the *in vivo *environment. This state likely reflects a fine balance between signals that maintain active consolidation and those that stimulate F-actin protrusions and branching. What are the mechanisms that control consolidation *in vivo*? In cultured neurons, consolidation is an active process, requiring Rho-kinase-mediated inhibition of actin polymerization at the growth cone neck [15], and calpain-mediated degradation of actin regulatory factors in the axon shaft [16]. Moreover, calpain inhibition in adult mice induces excess dendritic branching in hippocampal neurons [16], supporting a role for this pathway *in vivo*. Although calpain activity can be induced by low protein kinase A levels in the axon shaft [16], the upstream signals that activate consolidation or that control protein kinase A levels are not known. Our results suggest that the stimuli triggering consolidation are not immediately active *in vivo*. RB central axons initially extend along neuroepithelial cells before contacting and fasciculating with each other [37]. Perhaps axons must reach a critical length and/or engage sufficient adhesive contacts with neuroepithelial cells to activate consolidation signals. Alternatively, contact with other central axons may stimulate consolidation. Our RB model will be useful for future studies to define the molecular signals that regulate consolidation of pioneering axons *in vivo*.

Axon initiation is an important first step in neuronal morphogenesis, yet few studies have examined this process *in vivo*. Our observations that F-actin protrusive activity and accumulation precede RB peripheral axon emergence are consistent with previous findings that remodeling of the actin cytoskeleton has a key role in neurite initiation *in situ *and *in vitro *[9,10]. The specific position of axon initiation also is critical for neuronal morphology and polarity *in vivo*. We found that although F-actin-based filopodial protrusions can occur all along the central axons, only those in a cellular compartment containing the basal cell body and proximal central axons typically lead to peripheral axon initiation, suggesting this compartment is uniquely competent to generate a peripheral axon. There are several potential mechanisms controlling this axon initiation site. For example, filopodia in the compartment near the cell body may be favored for microtubule invasion because of better access to the microtubule organizing center. Indeed, both the formation of F-actin-based filopodia and their invasion by microtubules are required for neurite initiation in cultured neurons [10,54]. In addition, the inherent apical-basal organization of the spinal neuroepithelium likely influences RB polarity and may thus define the basal position of peripheral axon initiation. Extracellular signals also likely play a role in determining the axon initiation site. Our finding that somite boundary regions are favorable for peripheral axon exit from the spinal cord suggests they may be a localized source of growth-promoting cues. Extracellular UNC-6/netrin defines axon initiation position in *C. elegans *by directing asymmetric activation of an actin-regulatory protein, MIG-10/lamellipodin [11]. However, little else is known about extracellular signals that determine axon positioning in any *in vivo *system. On the whole, the precise location of axon initiation likely involves coordinated activity of external signals that promote and position outgrowth as well as intrinsic regulatory mechanisms that lead to localized remodeling of the cytoskeleton.

In addition to initiating axons at the correct position, neurons must regulate the number of axons they extend. In a subset of our imaging experiments, we observed transient initiation of multiple peripheral neurites from the basal cell body or central axons, all but one of which retracted after the successful axon exited the spinal cord. One potential explanation for this behavior is that axons encounter a signal upon spinal cord exit that suppresses supernumerary peripheral axons. Spinal cord exit is an important transition in the peripheral axon pathway, marked by a dramatic change in growth cone size and behavior. As they exit, peripheral axons must grow through the basal lamina, a layer of extracellular matrix enriched in laminin, which is a well-known regulator of axon outgrowth and guidance (for example, [55-61]). In zebrafish *bashful (bal)/laminin-α1 *mutants, supernumerary axons initiate from ectopic positions in midbrain neurons, suggesting laminin could act to regulate axon number or polarity [60,61]. Although peripheral RB axon trajectories were normal in *bal *embryos, their arbors appeared denser [60,61], suggesting they could have supernumerary peripheral axons. However, individual neuron morphologies have not been examined in these mutants. The extracellular matrix has key roles in regulating cell motility in multiple cell types, and thus is likely to influence both axon number and axon positioning.

Our live imaging has also provided insight into mechanisms underlying axon branching *in vivo*. Axon branches can form by two modes: growth cone bifurcation or interstitial collateral formation from the axon shaft [2,6,17]. We found that one of the first steps in peripheral axon branching is sustained F-actin accumulation, which is consistent with previous F-actin imaging during interstitial axon branching *in vitro *[26]. Several studies of other neuron types have shown that interstitial branching begins by formation of filopodia along the axon shaft (reviewed in [19]). Moreover, previous work showed that in cultured neurons filopodia are preceded by F-actin patches [15,62,63] that form in association with localized microdomains of phosphatidylinositol-3,4,5-triphosphate (generated by phosphoinositide 3-kinase) [62]. After the formation of F-actin accumulations and filopodia, microtubules must invade the filopodia for branch maturation [6,19]. Microtubule invasion requires local microtubule severing [64,65], dynamic instability [26], and transport of short microtubules into the branch [66]. Moreover, interactions between actin and microtubules are required for branch development [19,26], suggesting localized F-actin accumulations have an important function in directing microtubule capture and branch formation.

Final branch morphology does not necessarily reflect how a branch formed. Thus, distinguishing between bifurcation versus interstitial branching mechanisms requires live imaging with high temporal resolution. Previous live imaging of axon branching *in vivo *in mammalian cortex [23], in zebrafish or *Xenopus *optic tectum [46,47], or of zebrafish trigeminal sensory axons [67] showed that interstitial branching appears to be the predominant mode, although occasional growth cone splitting was observed in some studies [46,67]. We found that both growth cone bifurcations and interstitial branches commonly occur in RB peripheral axons as they arborize *in vivo*. RB neurons may employ bifurcation to lay out their overall arbor territory, and interstitial branching to fully arborize and fill the territory. In addition, we found that the two branching modes occur simultaneously within one neuron. In contrast, DRG sensory neurons, which serve a function analogous to RBs, display bifurcation and interstitial branching at distinct stages of their outgrowth [20,21], and recent evidence suggests the two branching modes may be controlled by different molecular mechanisms [6,20]. It will be important to determine the extent to which these branching modes employ common versus distinct molecular mechanisms. Multiple extracellular cues have been identified that can promote axon branches in other systems (reviewed in [2,6]). To date, the only branching factor identified that affects RB axons is Slit2, which can promote excess secondary branching of peripheral RB arbors when overexpressed [68]. The ability to combine live imaging with molecular manipulation makes the zebrafish RB model ideal for future studies to determine mechanisms of axon branching and the molecular differences in bifurcation versus interstitial branching.

### LIM-HD transcription factor activity differentially regulates RB axons

The importance of transcriptional regulation in axon development has only recently come to light, and little is known about the molecular and cellular steps between transcription and axon behavior. Previous studies implicated LIM-HD transcription factors in RB peripheral axon formation [39,40]. Here we show that these transcription factors function in several cell motility processes during RB morphogenesis, and we show how they differentially affect peripheral versus central axon behavior. LIM-HD activity is required for the initiation and outgrowth of the peripheral axon but not the central axons. Although most RB neurons did not initiate a peripheral axon in DN-CLIM embryos, F-actin accumulations and filopodia still formed in these neurons, suggesting LIM-HD activity does not affect the actin remodeling underlying axon initiation. Instead, our results suggest that LIM-HD activity regulates a subsequent step in axon development, perhaps controlling the ability of microtubules to invade filopodia and/or mediating interactions between the microtubule and actin cytoskeleton. Furthermore, our finding that F-actin accumulations and initiating neurites can form in ectopic apical locations on the RB cell body in DN-CLIM embryos suggests that LIM-HD activity contributes to the basal positioning of peripheral axons. The fact that DN-CLIM specifically affects peripheral and not central axon initiation suggests that distinct mechanisms may direct initiation of these two axon types.

DN-CLIM affects not only peripheral axon initiation, but also secondary branching in the periphery. This finding suggests that LIM-HD transcription factor activity could control multiple processes that occur at different stages of RB development. Alternatively, axon initiation and branching may be variations of the same process and may share similar underlying mechanisms mediated by LIM-HD activity. Indeed, in many cases the peripheral axon forms as a branch from the central axon. Both axon initiation and branching require precise control over actin rearrangement and microtubule invasion into the developing branch [4-8,26,54]. Moreover, several extracellular signals, such as netrin [11,25], are known to stimulate both processes in other systems. Axon branching and the underlying cytoskeletal changes are influenced both by branch promoting cues [2,6,18] and by suppressive signals that prevent ectopic branching [16,69]. LIM-HD activity could potentially modulate axon responses to either branch-stimulating or branch-suppressing signals.

LIM-HD transcription factor activity also differentially affects the motility of RB axons during outgrowth. In contrast to peripheral axons, whose growth was slowed by DN-CLIM, central axon growth rates were increased when LIM-HD activity was disrupted. This result could indicate that LIM-HD activity has a specific function in suppressing central outgrowth. Alternatively, the change in central axon growth rate may result from a general increase in available cellular resources when a peripheral axon does not extend. Consistent with the former hypothesis, differential neurite outgrowth ability of cultured *Xenopus *spinal neurons was shown to be mediated by an active intracellular signaling mechanism, and not simply by limited cellular resources [70]. Although we cannot distinguish between these possibilities, our results support the idea that an intrinsic transcriptional mechanism can selectively regulate the behavior of two axons from a single neuron.

How might LIM-HD transcription factors control the differential behavior of central versus peripheral axons? One possibility is that LIM-HD transcription factors regulate differential localization or trafficking of receptor or signaling molecules to one RB axon versus the other. Some evidence exists for molecular differences between central and peripheral RB axons. For instance, the cell surface glycoprotein TAG-1 (transiently expressed axonal glycoprotein 1)/Contactin-2 is required specifically for central axon extension, while extracellular Sema3D is repulsive to peripheral but not central axons [38]. In *Xenopus *RB neurons, focal adhesion kinase, which regulates adhesions and F-actin dynamics, is required for peripheral but not central RB axon growth [71]. To date, few downstream targets of LIM-HD transcription factors have been identified in zebrafish: *plexinA4 *(a semaphorin receptor) [68], *dpysl3/crmp4 *(a semaphorin signaling component) [72], and *contactin-1 *[73]. Dpysl3 cooperates with Sema3D to regulate peripheral axon outgrowth [72], PlexinA4 plays a role in mediating Slit2-induced secondary branching of peripheral RB axons [68], and contactin-1 functions in regulating *Xenopus *RB axon fasciculation [74]. These latter processes - branching and contact-mediated axon behaviors - represent key differences between central and peripheral axons, further supporting the idea that LIM-HD transcription factors control differential behavior of the axons. There are likely multiple downstream genes regulated by LIM-HD transcription factors that function together to control axon behavior. Indeed, inhibition of any one of the known target genes does not phenocopy the DN-CLIM effect, consistent with this idea. Future work to identify the essential downstream targets of LIM-HD transcription factors, and how they cooperate in RB neurons will be critical for understanding mechanisms of transcriptional regulation of neuronal morphogenesis.

## Conclusions

Our live imaging experiments provide insight into the cell motility processes underlying neuronal morphogenesis *in vivo*. We characterize cell behaviors and the dynamics of F-actin distribution during axon initiation, consolidation and branching in the natural *in vivo *environment. Our results demonstrate that LIM-HD transcription factor activity differentially regulates the motile behavior of two axons from one neuron. In addition, we show that LIM-HD activity does not affect F-actin protrusive activity or the ability of F-actin to accumulate during axon initiation and branching, but may have a role in peripheral axon positioning. The zebrafish RB model will be ideal for future studies to investigate molecular mechanisms that control these processes *in vivo*.

## Materials and methods

### Animals

Adult zebrafish (*Danio rerio*) were maintained in a laboratory breeding colony on a 14:10 h light:dark cycle. Wild-type AB strain and transgenic *Tg(-3.1ngn1:gfp) *(provided by U Strähle) [43] or *Tg(-3.1ngn1:gfp-caax) *lines with labeled primary sensory neurons were used for experiments. Embryos were raised at 23 to 28.5°C and staged as described previously [75]. Animals were handled in accordance with guidelines set forth by NIH and IACUC. Our animal use protocol was approved by the University of Wisconsin Animal Care and Use Committee.

### DNA constructs

DNA expression constructs were generated with the Multisite Gateway^® ^Cloning System (Invitrogen, Carlsbad, CA, USA) using zebrafish-compatible Tol2 transposon vectors [76-78] (Tol2 kits provided by K Kwan, C-B Chien and N Lawson), as described [77].

To drive expression in Rohon-Beard neurons, a 3.1-kb *cis*-regulatory element from the zebrafish *neurogenin1 *gene (*-3.1ngn1*) [43] was subcloned from *pCS2:ngn3.1-GFP *(provided by U Strähle) into the SpeI/MslI sites of *p5E-MCS *(Tol2 kit). The resulting construct, *p5E-ngn(-koz)*, lacks the kozak translational start site sequence, and was subsequently used to generate all expression vectors in this study.

For transgenesis and cell labeling, we used fluorophores GFP or TagRFP (Axxora LLC, San Diego, CA, USA) fused to a CAAX box prenylation sequence, which targets proteins to the plasma membrane. Tol2 kit middle entry vectors [77] were used to generate expression vectors. Two-way gateway recombination cloning using *pTolDestR4-R2pA *[78] generated RB-specific expression constructs *pEXP-3.1ngn1:gfp-caax *or *pEXP*-*3.1ngn1:tagrfp-caax*, referred to as GFP-CAAX and TagRFP-CAAX, respectively.

For F-actin labeling, the F-actin-binding calponin homology domain of utrophin with amino-terminal mCherry was amplified from *pCS2+mCh-utrCH *[48] (gift from B Burkel and W Bement) and cloned by Gateway recombination into *pDONR221 *(Invitrogen). The resulting middle entry vector (*pME-mCherry-UtrCH*) was subsequently used in a two-way gateway recombination reaction to generate *pEXP-3.1ngn1:mCherry-UtrCH *(referred to as mCh-UtrCH).

### RNA synthesis

5'-Capped RNA was synthesized from template DNA *in vitro *using the mMessage mMachine kit (Ambion, Austin, TX, USA). Tol2 transposase RNA was generated using plasmid *pCS2FA-transposase *DNA (Tol2 kit), as described [77]. DN-CLIM RNA was generated using plasmid *pCS2+DN-CLIM *DNA (gift from I Bach), as described [39].

### Stable transgenesis

A transgenic line, *Tg*(*-3.1ngn1:gfp-caax*), expressing membrane-targeted GFP in all RB neurons was generated by co-injecting 50 pg (*pEXP-3.1ngn1:gfp-caax*) plasmid DNA along with 25 pg of Tol2 transposase mRNA into wild-type AB embryos at the one-cell stage. Injected G0 embryos were screened by epifluorescence and raised to adulthood. F1 progeny were screened for stable transmission of the *gfp-caax *transgene and GFP-positive embryos were raised to establish the line. F2 or F3 embryos were used for experiments.

### Cell and F-actin labeling by transient transgenesis

Membrane-targeted fluorophores and mCh-UtrCH were used to visualize cell motility and F-actin, respectively, in developing RB neurons. Injection of plasmid DNA at the one-cell stage results in transient mosaic expression. Embryos were microinjected with 10 to 25 pg of DNA encoding GFP-CAAX, TagRFP-CAAX or mCh-UtrCH in RB-specific expression vectors. Embryos containing individually labeled RB neurons were dechorionated and sorted under epifluorescence illumination using a Nikon AZ100 dissecting microscope equipped with a 4× objective (at 40× magnification). RB neurons in the central region of the trunk (somites 3 to 14) were selected for imaging.

### DN-CLIM expression

LIM-HD activity was disrupted by ubiquitous expression of a dominant negative cofactor of LIM (DN-CLIM) [39]. Approximately 150 pg of DN-CLIM mRNA was injected into wild-type or transgenic embryos at the one-cell stage. Embryos were sorted at approximately 16 hpf for DN-CLIM-associated eye and midbrain-hindbrain boundary phenotypes [39], and those with morphological defects were selected for further analysis. Efficacy of DN-CLIM was assessed after imaging by examining all RB neurons and confirming the peripheral RB axon outgrowth phenotype.

### Fixed embryo imaging

To analyze mature RB morphology and the extent of peripheral axon branching, selected embryos with individually labeled RB neurons were raised to 24 hpf. Embryos were fixed in 4% paraformaldehyde overnight at 4°C and washed stepwise into 70% glycerol in phosphate-buffered saline. Embryos were whole mounted and imaged on an Olympus Fluoview1000, IX81 confocal microscope equipped with a 60× oil immersion objective (NA 1.35).

### Live embryo imaging

Embryos were anesthetized with 0.02% 3-amino benzoic acid ethylester (tricaine) then mounted in 1% low melting point agarose in 10 mM HEPES-buffered E3 embryo medium, as described [42]. Images were captured using an Olympus Fluoview1000, IX81 confocal microscope equipped with a 60× oil immersion objective (NA 1.35).

Z-stacks of 0.5- to 2-μm step sizes encompassing a total of approximately 25 to 30 μm into the trunk were taken of lateral or dorsal-lateral mounts to visualize the RB peripheral and central axon pathways. For time-lapse imaging, embryos ranged in age from 16 to 19 hpf at the beginning of the experiment and were imaged for durations of 2 to 8 hours at 28°C, with Z-stacks captured at 1 to 2 minute intervals.

### Image analysis and quantifications

Images were processed and analyzed using Volocity software (Perkin Elmer, Waltham, MA, USA), and figures assembled with Adobe Photoshop (Adobe Systems, Inc., San Jose, CA, USA). Movies were processed and built with Volocity and ImageJ software, and are played at a rate of 4, 6 or 9 frames per second depending on the time intervals used during acquisition. In figures with time-lapse images, time is displayed relative to the first image in the series shown.

Axonal growth rates were calculated as distance over time, and are reported as μm/h. Distance was determined by measuring axon length in XYZ dimensions at two time points of axon extension and calculating their difference. Only axons that extended through the field of view for at least 20 minutes were measured. Central axon lengths were measured from their initiation site on the cell body to the growth cone tip. To compensate for drift during imaging, fixed embryonic features, such as RB cell body or somite boundary position, were used as positional landmarks. Peripheral axon lengths were measured from their initiation site (on the cell body or central axon) to the leading growth cone of the peripheral arbor. For peripheral axons, growth rates were measured starting at the time that the axon reaches the skin and grows ventrally while branching.

Peripheral branching was measured by counting the number of axon termini per axon arbor/neuron in mosaically labeled or transgenic embryos, and expressed as number of axon tips per neuron. The extent of peripheral RB axon branching was assessed in both fixed and live wild-type embryos, and exclusively in live DN-CLIM embryos. To remain consistent, only peripheral axon arbors that extended past the horizontal myoseptum (approximately 80 μm in the ventral direction) were quantified for extent of branching, generally between 21 and 24 hpf depending on the anterior-posterior axial position of the RB neuron within the trunk.

F-actin protrusive activity was measured by counting the number of F-actin-containing filopodia that formed along RB central axons during the time when a peripheral axon formed, or would be expected to form in the case of DN-CLIM-expressing embryos (phase II). Filopodia were defined as visible protrusions, labeled with mCh-UtrCH, with a length of at least 1.5 μm, and were counted over a 20-minute time window. Protrusive activity is reported as number of filopodia per hour.

Statistical and graphical analyses were performed using Microsoft Excel software. Axon growth rates, extent of axon branching, and protrusive activity are reported as mean ± standard error of the mean. Statistical significance was assessed using a two-tailed *t*-test.

## Abbreviations

DN-CLIM: dominant negative cofactor of LIM; F-actin: filamentous actin; GFP: green fluorescent protein; Hox: homeobox; hpf: hours post-fertilization; LIM-HD: LIM homeodomain; RB: Rohon-Beard; TagRFP: Tag red fluorescent protein; UtrCH: utrophin calponin homology.

## Competing interests

The authors declare that they have no competing interests.

## Authors' contributions

EA and NA did the experiments and data analysis, and contributed to experimental design. MH supervised the experiments and contributed to experimental design. All three authors participated in writing the manuscript. All authors read and approved the final manuscript.

## Supplementary Material

Additional file 1 **Movie of RB central axon development**. Movie of a *-3.1ngn1:TagRFP-CAAX*-expressing RB neuron in Figure 1D. Images were acquired every 1:30 to 2:15 minutes over 224.5 minutes between 18 and 21.5 hpf. Click here for file

Additional file 2 **Movie of RB peripheral axon development**. Movie of two *-3.1ngn1:GFP-CAAX*-expressing RB neurons in Figure 2B. Images were acquired every 1:15 to 1:30 minutes over 193.5 minutes between 18 and 21 hpf.
Click here for file

Additional file 3 **Movie of F-actin distribution in a developing wild-type RB neuron**. Movie of a *-3.1ngn1:mch-utrCH*-expressing RB neuron in Figure 3A. Images were acquired every minute over 240 minutes between 17 and 21 hpf. A peripheral axon grows out of the focal plane during part of the movie.
Click here for file

Additional file 4 **Movie of DN-CLIM-expressing embryo in which RB peripheral axon fails to initiate**. Movie of a *-3.1ngn1:TagRFP-CAAX*-expressing RB neurons in Figure 5A. Images were acquired every minute over 334 minutes between 18 and 23.5 hpf.
Click here for file

Additional file 5 **Movie of a DN-CLIM-expressing embryo with aberrant peripheral axon outgrowth**. Movie of a *-3.1ngn1:TagRFP-CAAX *expressing RB neurons in Figure 5D. Images were acquired every minute over 340 minutes between 18 and 23.5 hpf. Click here for file

Additional file 6 **Movie of F-actin distribution in a DN-CLIM-expressing embryo in which a peripheral axon fails to initiate**. Movie of a *-3.1ngn1:mch-utrCH*-expressing RB neuron in Figure 6A. Images were acquired every 1:30 to 2:00 over 302 minutes between 18 and 23 hpf.
Click here for file

Additional file 7 **Movie of F-actin distribution in a DN-CLIM-expressing embryo in which aperipheral axon initiates, but fails to extend**. Movie of a *-3.1ngn1:mch-utrCH*-expressing RB neuron in Figure 6B. Images were acquired every minute over 90 minutes between 18 and 19.5 hpf.
Click here for file
